# Oxygen vacancy-driven bandgap tuning and ultrafast laser performance in Bi_2_O_2_Se

**DOI:** 10.1515/nanoph-2025-0274

**Published:** 2025-09-12

**Authors:** Qingling Tang, Zhongben Pan, Zeshang Ji, Hongwei Chu, Han Pan, Dechun Li

**Affiliations:** School of Information Science and Engineering, Key Laboratory of Laser and Infrared System of Ministry of Education, 12589 Shandong University, Qingdao 266237, China

**Keywords:** Bi_2_O_2_Se, oxygen vacancy defect, first-principles calculations, 1 μm laser, nonlinear optics

## Abstract

Bi_2_O_2_Se, with its excellent air stability, high mobility, and tunable bandgap, shows great potential for photonic modulators. This study proposes vacancy engineering as an effective method to refine its optical properties and enhance the nonlinear response. The optical properties of Bi_2_O_2_Se modified by oxygen vacancy defects were systematically investigated through theoretical simulations and experimental methods. Oxygen vacancies enhance photon–material interactions by introducing intermediate energy levels, altering the electronic structure, reducing the bandgap, inducing a redshift in the linear absorption spectrum, and forming a new absorption band near 1 μm. Argon annealing increased the concentration of oxygen vacancies, and the experimental absorption spectra showed excellent agreement with theoretical predictions. To evaluate the impact of oxygen vacancies on the nonlinear optical response, Bi_2_O_2_Se before and after annealing was employed as a saturable absorber in Q-switched and mode-locked lasers. The annealed Bi_2_O_2_Se exhibited a 4.42-fold increase in peak power, a 115.9 fs reduction in pulse width, and a 2.36 nm expansion in 3 dB spectral width. These findings indicate that vacancy engineering is a direct and effective strategy for optimizing the nonlinear optical properties of Bi_2_O_2_Se, which can contribute to advanced photon applications.

## 1 Introduction

Ultrafast laser technology has been widely applied in scientific research, precision machining, and biomedical fields in recent years [1], [2], [3], [4], [5]. Femtosecond laser technology enables high-resolution visualization of biological dynamics through nonlinear optical imaging techniques (e.g., two-photon fluorescence and Harmonic Generation microscopy), while simultaneously driving ultrashort-pulse systems to achieve high-power precision processing in industrial applications with minimal thermal damage [6], [7]. Meanwhile, nonlinear optical materials have demonstrated significant potential in cancer photothermal therapy, medical imaging enhancement, high-efficiency photovoltaic conversion, and novel energy storage systems. By developing novel functional crystals and tailoring their optical response properties, researchers have achieved breakthrough progress in deep-tissue imaging, ultrafast optical switching devices, and photoelectric energy conversion applications [8], [9], [10], [11], [12], [13], [14]. Since the emergence of broadband tunable laser crystals and mode-locking technology in the 1980s, femtosecond laser technology has achieved rapid development [15], [16], [17], [18]. In mode-locking technology, the stable generation of high-quality ultrashort pulses is crucial for achieving high-precision applications, and the performance of the saturable absorber (SA) as a core component directly determines the output characteristics of the laser [19]. To date, semiconductor saturable absorber mirrors (SESAMs) are undoubtedly one of the most successful types of SAs. However, the high cost, narrow operating bandwidth, low damage threshold, and complex manufacturing process of SESAMs limit their application in certain fields [20], [21], [22]. Therefore, the development of novel saturable absorbers with superior performance has become a significant focus in the study of ultrafast optical phenomena in fiber laser systems [23], [24], [25], [26], [27].

In recent years, Bi_2_O_2_Se has garnered significant attention in the field of ultrashort pulsed lasers owing to its exceptional ultra-broadband nonlinear modulation characteristics and high nonlinear absorption coefficient. Bi_2_O_2_Se is a typical bismuth-based oxyselenide material belonging to the orthorhombic crystal system, where each oxygen atom is edge-shared with four bismuth atoms to form Bi_4_O tetrahedra. These tetrahedra are edge-connected to form positively charged [Bi_2_O_2_]_*n*_^2*n*+^ layers, alternating with negatively charged [Se]_*n*_^2n−^ layers, held together by relatively weak electrostatic forces (Figure S1) [28], [29]. This unique electronic structure contributes to improving the performance and optimizing the functionality of nonlinear optical devices. Furthermore, its narrow bandgap (approximately 0.8 eV) makes it highly promising for applications in infrared photodetection [30], [31]. Bi_2_O_2_Se was first utilized as a SA in 2019 to achieve a 1.55 μm mode-locked erbium-doped fiber laser and a 2 μm Q-switched Tm–Ho codoped fiber laser, with pulse durations of 6.1 ps and 4.1 μs, respectively [32]. Its potential as an optical modulator in the near-infrared spectral range was preliminarily verified. To further exploit its properties, research focus has gradually shifted from the direct application of Bi_2_O_2_Se to performance modulation through defect engineering. This strategic pivot is driven by the proven efficacy of defect engineering in enhancing nonlinear optical responses across diverse material systems [33], [34], [35], [36]. In 2022, Bi_2_O_2_Se nanosheets were employed as SAs in 1 μm fs solid-state lasers, where plasma treatment was used to simultaneously modulate oxygen and selenium defect states, achieving an average output power of 665 mW and a pulse duration of 266 fs. [37]. However, the practical applicability of the plasma treatment method is limited by its high process complexity and challenges in precisely controlling defect types. Furthermore, systematic theoretical studies on the impact of vacancy defects particularly oxygen vacancies (which are common point defects in oxide materials) on the optical properties of Bi_2_O_2_Se remain scarce. Oxygen vacancies can induce localized lattice distortions, leading to changes in the electronic structure and optical properties of the material, which significantly influence its nonlinear optical characteristics [38], [39], [40]. However, these mechanisms in Bi_2_O_2_Se are not yet fully understood, highlighting the need for further in-depth investigation.

In this study, the influence of oxygen vacancies in Bi_2_O_2_Se on its optical properties is systematically investigated for the first time by combining first principles calculations, annealing modulation, and experimental validation. First, first principles calculations are employed to analyze changes in Bader charge distribution, energy band structure, density of states, dielectric constant, and absorption spectrum of Bi_2_O_2_Se in the presence of oxygen vacancy defects. The results reveal that the introduction of oxygen vacancies significantly enhances the material’s light absorption at 1 μm, and the underlying mechanism is thoroughly explored. To validate these theoretical calculations, the oxygen vacancy content in Bi_2_O_2_Se is modulated through argon annealing, and the absorption spectra are measured and compared with the calculated results, confirming the accuracy of the theoretical predictions. Furthermore, the saturable absorption properties of Bi_2_O_2_Se before and after annealing are evaluated using intensity scanning (I-scan) and open-aperture Z-scan (OA Z-scan) techniques. Finally, Bi_2_O_2_Se with varying oxygen vacancy contents are applied in 1 μm fiber lasers, achieving passive Q-switching and mode-locking operations to generate ultrashort pulses. This study focuses on the oxygen vacancy engineering to regulate the nonlinear optical response and ultrashort pulse laser performance of Bi_2_O_2_Se and combines the theory and experiments closely to reveal the important role of oxygen vacancies in Bi_2_O_2_Se, which provides insights into the mechanism by which its physical properties are modulated and support its potential application in high-performance lasers.

## 2 Results and discussion

### 2.1 Structural properties and defect formation energy

Figure S1(a) shows the optimized conventional crystal structure of Bi_2_O_2_Se (1 × 1 × 2). According to previous calculations and experiments, the lattice constants of the Bi_2_O_2_Se unit cell are *a* = *b* = 3.89 Å, *c* = 12.16 Å [41]. Thus, it can be inferred that the lattice constants of the 1 × 1 × 2 structure are *a* = 3.89 Å, *b* = 7.78 Å, and *c* = 12.16 Å. The lattice constants after structural optimization are shown in Table 1, consistent with the expected calculations, confirming the reliability of the optimization method.

**Table 1:** Lattice constants, formation energies, and bond lengths (1 × 1 × 2).

	Bi_2_O_2_Se	Bi_2_O_1.5_Se
*a* (Å)	3.93	3.92
*b* (Å)	7.85	7.81
*c* (Å)	12.39	12.35
Bi1–Bi2 (Å)	3.93	3.69
Bi3–Bi4 (Å)	3.99	3.92
Oi1–O2 (Å)	3.91	3.62

In Bi_2_O_2_Se, all oxygen sites are equivalent; thus, Bi_2_O_1.5_Se with vacancy defects is obtained by removing two oxygen atoms from the system, as illustrated in Figure S1(b). After optimization, the lattice constants of Bi_2_O_1.5_Se are 3.92 Å, 7.81 Å, and 12.35 Å, respectively. The structural optimization results show that the distances between Bi1–Bi2, Bi3–Bi4, and O–O around oxygen vacancies exhibit a decreasing trend. This is because the oxygen vacancies introduce positive charges, which attract surrounding atoms and shorten bond lengths, resulting in an overall contraction of the lattice constant *c*.

### 2.2 Bader charge analysis

**Figure 1:**
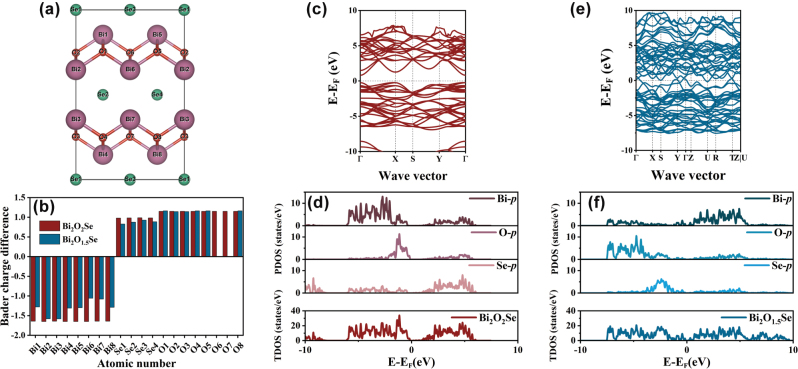
Theoretical calculation. (a) Bi_2_O_2_Se side view. (b) Bader charge difference. (c) Bi_2_O_2_Se energy bands. (b) Bi_2_O_2_Se density of states. (c) Bi_2_O_1.5_Se energy bands. (d) Bi_2_O_1.5_Se density of states.

The creation of oxygen vacancies leads to charge redistribution in the surface and subsurface layers, motivating a detailed investigation of their effect on the Bader charge. As shown in Figure 1(b), the horizontal axis corresponds to the atomic number in Figure 1(a), while the vertical axis represents the Bader charge difference, reflecting the electron transfer process within the system. In Bi_2_O_1.5_Se, oxygen vacancy defects act as electron donors, resulting in significant changes in the electronic structure of the material. Specifically, the presence of O6 and O7 vacancies reduces electron loss from nearby Bi atoms, increasing charge localization energy and limiting the amount of charge acquired by adjacent Se atoms. In contrast, at O2 and O3 sites, which are farther from the oxygen vacancies, gain fewer electrons compared to Bi_2_O_2_Se. This suggests that the formation of oxygen vacancies increases the charge on Bi atoms, redistributing the electronic energy levels. These changes may shift the material’s energy band structure and alter its electronic and optical properties.

### 2.3 Energy band structure and density of states

The introduction of oxygen vacancies in Bi_2_O_2_Se disrupts the original symmetry, leading to charge redistribution and consequently affecting the material’s band structure [42]. For Bi_2_O_2_Se, as shown in Figure 1(c) and (d), it is an indirect bandgap semiconductor with a bandgap value of 1.04 eV, consistent with previous calculations [43]. The valence band maximum (VBM) primarily arises from the hybridization of Bi 6*p*, O 2*p*, and Se 4*p* orbitals, while the conduction band minimum (CBM) is mainly contributed by the Bi 6*p* orbital. Upon introducing oxygen vacancies, as shown in Figure 1(e) and (f), the band structure and density of states (DOS) undergo significant changes. Although the defected Bi_2_O_1.5_Se retains its indirect bandgap characteristics, the VBM shifts from the Gamma point (Γ) to the *Y* point, the CBM shifts from the Gamma point to the *U* point, and generates new defect levels within the forbidden band, reducing the bandgap and resulting in a transition to semimetallic behavior, which is consistent with the experimentally observed properties of oxygen vacancy defect nanosheets [44]. Further analysis of the projected density of states (PDOS) reveals that the impurity levels introduced by oxygen vacancies are primarily contributed by Bi 6*p* orbitals, indicating that Bi 6*p* orbitals are the main source of defect states within the bandgap. The presence of oxygen vacancies enhances interactions between defects, increasing band dispersion and amplifying the DOS associated with oxygen vacancies. They demonstrate that oxygen vacancies play a crucial role in tuning the electronic structure of Bi_2_O_2_Se and provide robust theoretical support for its potential applications in functional devices.

### 2.4 Complex dielectric constant and absorption spectra

**Figure 2:**
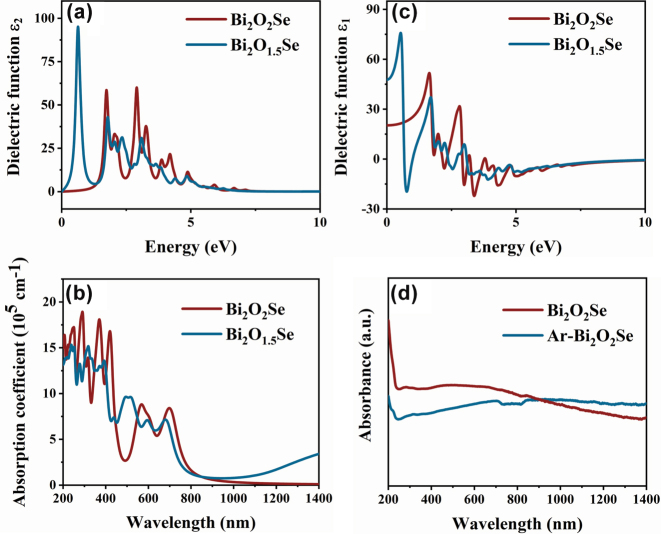
Dielectric constants and optical properties of Bi__2__O__2__Se and Bi__2__O__1.5__Se. (a) Real part of dielectric constant. (b) Imaginary part of dielectric constants. (c) Calculated absorption coefficient. (d) Test absorption spectrum.

As a promising material for optoelectronic devices, it is essential to gain a deep understanding of the optical properties of Bi_2_O_2_Se. To investigate the optical performance of Bi_2_O_2_Se with oxygen vacancies, this study calculated its complex dielectric function $\varepsilon \left(\omega \right)=\varepsilon_{1}\left(\omega \right)+i\varepsilon_{2}\left(\omega \right)$ [45], where the imaginary part $\varepsilon_{2}\left(\omega \right)$ arises from transitions between occupied and unoccupied states and is closely related to the electronic band structure of the material. The formula is:

(1) $$\begin{array}{ll} \varepsilon_{2}\left(\omega \right) & =\left(\frac{4^{2}\pi^{2}e^{2}}{m\omega^{2}}\right)\underset{i,j}{\sum }{\int \left\langle i\left|M\right|j\right\rangle }^{2}f_{i}\left(1-f_{i}\right)\\ & \;\times \delta \left(E_{j,k}-E_{i,k}-\omega \right)d^{3}k \end{array}$$

where *e* is the electron charge, *m* is the free electron mass, *ω* is the incident photon frequency, M is the dipole moment array, *f*_*i*_ is the Fermi distribution function for the *i*th state with wave function vector *k*, and *i* and *j* are the initial and final states, respectively. According to the Kramers–Kronig transform, the real part *ɛ*_1_(*ω*) can be derived from the imaginary part *ɛ*_2_(*ω*), which is:

(2) $$\begin{array}{c} \varepsilon_{1}\left(\omega \right)=1-\frac{2}{\pi }P\int_{0}^{\infty }\frac{\omega^{\prime }\varepsilon_{1}\left(\omega^{\prime }\right)d\omega^{\prime }}{\left(\omega^{\prime 2}-\omega^{2}\right)} \end{array}$$

where *P* denotes the principal value of the integral. Figure 2(a) and (b) illustrate the real and imaginary parts of the dielectric function for Bi_2_O_2_Se and Bi_2_O_1.5_Se, respectively. From the real part, the static dielectric constants of the two materials are 20.28 (Bi_2_O_2_Se) and 47.75 (Bi_2_O_1.5_Se). Materials with high dielectric constants typically exhibit strong nonlinear optical responses, suggesting that increasing the oxygen vacancy concentration in Bi_2_O_2_Se may enhance its potential for optical applications. The peaks in the imaginary part provide information about the electronic excitation. The first absorption peak is closely related to electronic transitions between occupied and unoccupied states near the Fermi level [46]. After the introduction of oxygen vacancies, Bi_2_O_1.5_Se exhibits a new absorption peak located at approximately 0.63 eV. The shift in the peak position corresponds to changes in defect energy levels within the band structure. Notably, the enhanced peaks in the low-energy region of the imaginary part indicate improved light absorption at longer wavelengths. Combining analyses of the band structure and DOS, it can be further concluded that the first absorption peak in Bi_2_O_2_Se corresponds to electronic transitions from Se 4*p* orbitals to Bi 6*p* orbitals, while in Bi_2_O_1.5_Se, the first absorption peak originates from transitions between Bi 6*p* orbitals. The increase in the peak value of the imaginary part indicates a higher interband transition probability, contributing to enhanced optical absorption of the material.

The absorption coefficient $\alpha \left(\omega \right)$ can be derived from the dielectric function:

(3) $$\begin{array}{c} \alpha \left(\omega \right)=\sqrt{2}\omega {\left[\sqrt{\varepsilon_{1}{\left(\omega \right)}^{2}+\varepsilon_{2}{\left(\omega \right)}^{2}}-\varepsilon_{1}\left(\omega \right)\right]}^{\frac{1}{2}} \end{array}$$

Figure 2(c) shows the absorption coefficients of Bi_2_O_2_Se and Bi_2_O_1.5_Se based on theoretical calculations. The results clearly demonstrate that the presence of oxygen vacancies leads to new absorption peaks in the long-wavelength region, consistent with previous theoretical analyses.

Additionally, the absorption peaks of Bi_2_O_2_Se and Bi_2_O_1.5_Se intersect near 850 nm, and beyond this point, the absorption of Bi_2_O_1.5_Se significantly surpasses that of Bi_2_O_2_Se. According to the calculations, in Bi_2_O_1.5_Se, the electron-rich environment around Bi atoms and the contribution of oxygen atoms to Bi atoms alter the DOS at the VBM, resulting in a reduced bandgap and broadened DOS. This change ultimately enhances light absorption, particularly in the low-absorption region, further expanding the material’s application potential. To validate the theoretical calculations, materials with higher oxygen vacancy concentrations, Ar-Bi_2_O_2_Se were prepared through argon annealing. And its ultraviolet-visible-near-infrared (UV–VIS–NIR) absorption spectra were measured (Figure 2(d)). The experimental results closely align with the theoretical calculations, confirming that oxygen vacancies effectively tune the optical properties of Bi_2_O_2_Se in the near-infrared region. During annealing, the increased concentration of oxygen vacancies further enhances the material’s optical absorption, demonstrating the critical role of oxygen vacancies in regulating material performance.

### 2.5 Material characterization

**Figure 3:**
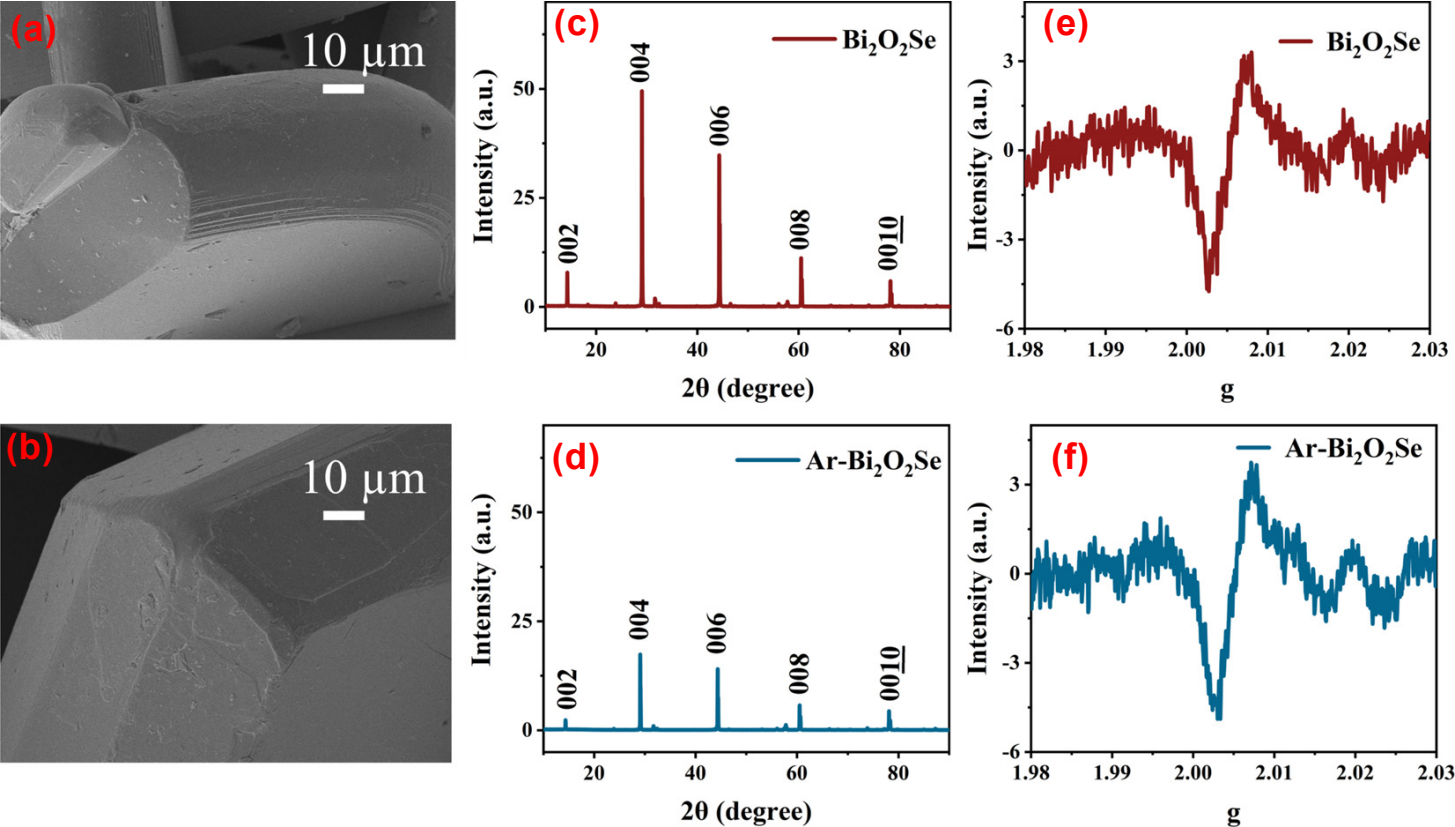
Morphology characterization. (a) Bi_2_O_2_Se SEM. (b) Ar-Bi_2_O_2_Se SEM. (c) Bi_2_O_2_Se XRD. (d) Ar-Bi_2_O_2_Se XRD. (e) Bi_2_O_2_Se EPR. (f) Ar-Bi_2_O_2_Se EPR.

We processed the data through argon annealing and referred to the annealed bismuth selenoxide as Ar-Bi_2_O_2_Se. The morphology of the material before and after annealing treatment using argon gas is shown in Figure 3(a) and (b). Scanning electron microscope (SEM) images show that the number of Bi_2_O_2_Se nanoparticles is reduced after annealing, but the average size of the large particles remains almost unchanged. This indicates that argon annealing has less effect on the grain morphology and size of the material. Figure 3(c) and (d) show the X-ray diffraction (XRD) results of the materials before and after annealing. Although the peak intensities are slightly reduced, the positions of the XRD peaks remain the same, indicating that the annealing has not changed the lattice constants or the symmetry of the crystal structure. Overall, the crystal structure remains stable during the annealing process and the crystal integrity of the material is well preserved.

In order to verify that annealing can modify the concentration of oxygen vacancies in Bi_2_O_2_Se, we performed an electron paramagnetic resonance (EPR) test, a highly sensitive technique for detecting unpaired electrons and thus characterizing oxygen vacancies in the material [47]. The sharp EPR signal detected at *g* = 2.005 indicates the presence of oxygen vacancies in the material both before and after annealing (Figure 3(e) and (f)). Quantitative analysis revealed a 13.33 % increase in peak intensity postannealing, with spin density rising from 1.691 × 10^11^ spins/mm^3^ to 1.926 × 10^11^ spins/mm^3^ (Δ = 2.35 × 10^10^ spins/mm^3^). This confirms that annealing can increase the oxygen vacancy content of Bi_2_O_2_Se.

### 2.6 Saturable absorption property

**Figure 4:**
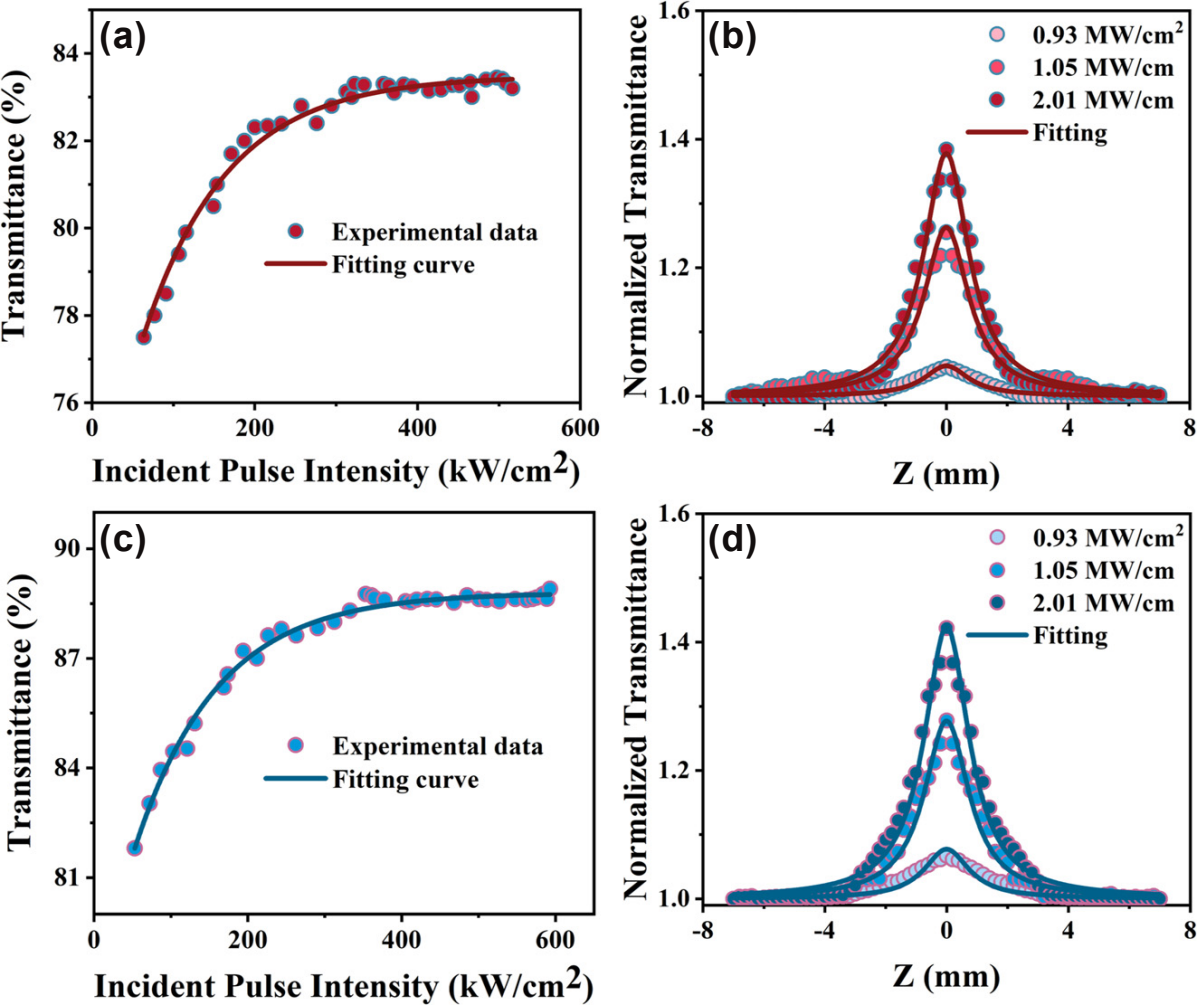
Characterization of nonlinear optical properties. (a) Nonlinear optical transmission versus the excitation intensity of Bi_2_O_2_Se at 1 μm. (b) Open-aperture Z-scan data and theoretically fitted curves of Bi_2_O_2_Se at 1 μm. (c) Nonlinear optical transmission versus the excitation intensity of Ar-Bi_2_O_2_Se at 1 μm. (d) Open-aperture Z-scan data and theoretically fitted curves of Ar-Bi_2_O_2_Se at 1 μm.

The saturable absorption properties of Bi_2_O_2_Se under 1 μm laser excitation were characterized using open-aperture (OA) Z-scan and I-scan techniques. The experiments employed excitation pulses with a pulse duration of 67.16 ns and a repetition rate of 4 kHz. Figure 4(a) and (b) illustrate the relationship between nonlinear transmittance and excitation intensity at 1 μm, it is clear that the transmittance increases significantly at the initial intensity and saturates at a relatively large intensity. The saturable absorption curve can be fitted by [48]

(4) $$\begin{array}{c} T=1-\Delta Rexp\left(\frac{-I}{I_{s}}\right)-\alpha_{ns} \end{array}$$

where *T* denotes the normalized transmittance, Δ*R* is the modulation depth, *I* is the incident light intensity, *I*_*s*_ is the saturated light intensity, and *α*_*ns*_ is the unsaturated loss. The results show that Bi_2_O_2_Se has a modulation depth of 5.7 %, which increases to 7.1 % after argon annealing (Ar-Bi_2_O_2_Se). This enhancement is attributed to the introduction of oxygen vacancies, which improve the material’s absorbing ability and modulate incident light, thereby increasing the modulation depth. Under excitation, the saturation intensities of Bi_2_O_2_Se and Ar-Bi_2_O_2_Se were measured as 103.42 kW cm^−2^ and 107.78 kW cm^−2^, respectively, while the unsaturated loss decreased from 16.5 % to 11.4 %. The higher saturation intensity of Ar-Bi_2_O_2_Se indicates greater stability under high-power laser conditions, and the lower unsaturated loss reflects reduced absorption losses at low power, ensuring efficient optical performance across a broad power range.

Nonlinear absorption coefficients were determined using OA Z-scan measurements (Figure 4(c) and (d)), which can be fitted by the following formula [49]:

(5) $$\begin{array}{c} T=\overset{\infty }{\underset{m=0}{\sum }}\frac{\left[-q_{0}\left(z,0\right)\right]}{{\left(m+1\right)}^{\frac{3}{2}}},\left(m\in N\right),q_{0}\left(z,0\right)=\frac{\beta_{\text{eff}}\cdot L_{\text{eff}}\cdot I_{0}}{1+\frac{z^{2}}{z_{0}^{2}}} \end{array}$$

where *T* and *z* are normalized transmittance and the relative distance of the sample on the *z*-axis, *L*_eff_ is the effective length of the sample, *I*_0_ is the maximum intensity, and *z*_0_ is the Rayleigh length. By irradiating the sample with laser pulses at 1 μm wavelength with incident intensities of 0.93 MW/cm^2^, 1.05 MW/cm^2^, and 2.01 MW/cm^2^, the measured nonlinear absorption coefficients were −0.03 cm/GW, −0.15 cm/GW, and −0.21 cm/GW for Bi_2_O_2_Se, and −0.04 cm/GW, −0.16 cm/GW, and −0.24 cm/GW for Ar-Bi_2_O_2_Se, respectively. The observation of saturable absorption stems from the Pauli exclusion principle: once the pertinent electron energy states are occupied under optical excitation, further carrier transitions are inhibited, resulting in absorption saturation [33]. In general, the enhanced modulation depth is primarily attributed to oxygen vacancy-induced localized electronic states within the bandgap. These defect states create additional energy levels that preferentially trap photogenerated electrons, suppressing direct carrier recombination. Simultaneously serving as intermediate energy levels that facilitate multiphoton transitions to reduce the excitation threshold and enhance the nonlinear absorption coefficient. The synergistic interplay between these electron-trapping and multiphoton transition mechanisms enables oxygen vacancies to optimize carrier relaxation pathways, ultimately yielding a 1.4 % improvement in modulation depth relative to nonannealed samples.

### 2.7 Q-switching

**Figure 5:**
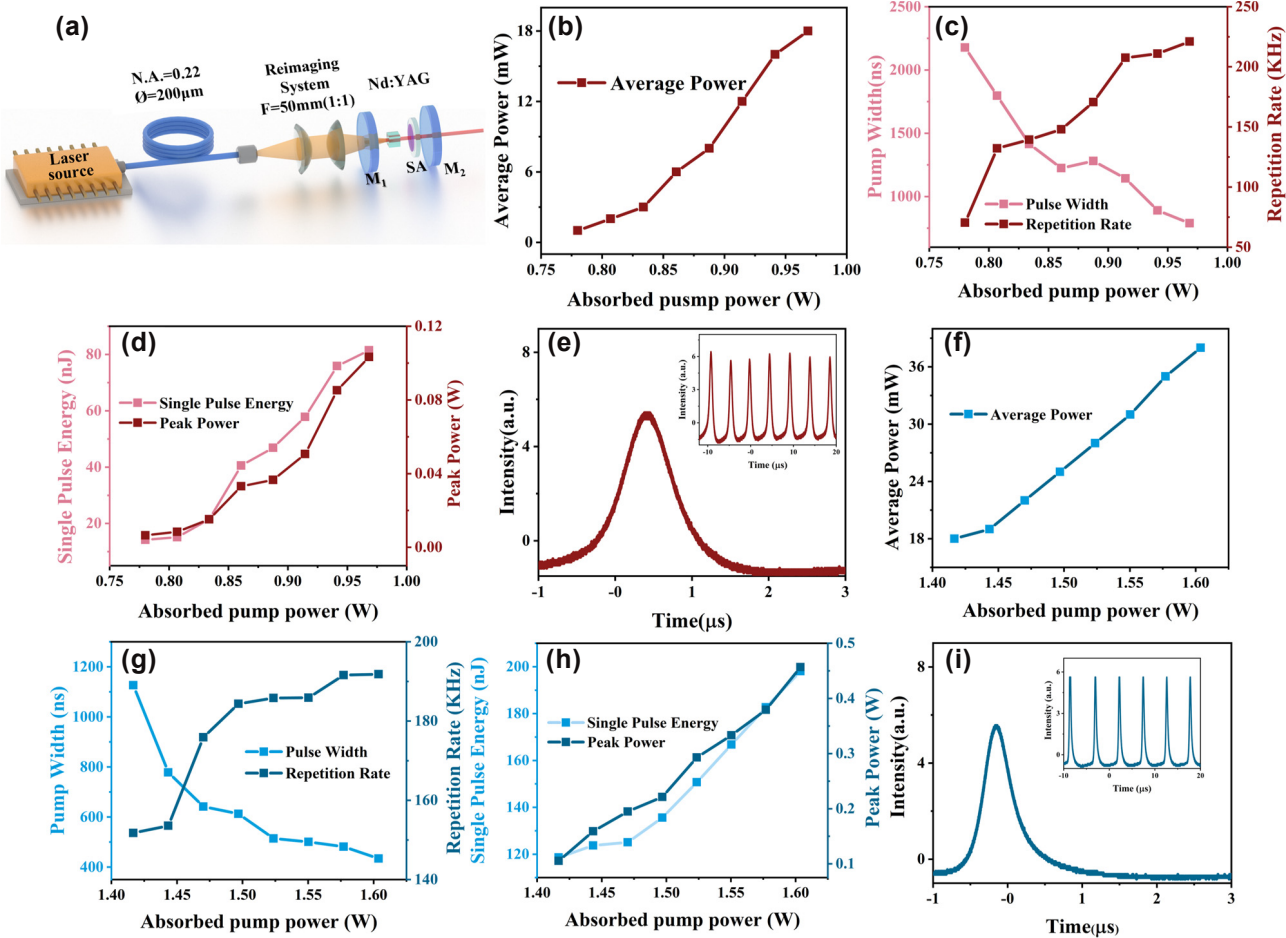
Experimental performances of the passively Q-switching lasers at 1.06 μm. (a) Schematic of the Q-switching laser cavity based on Bi_2_O_2_Se different defect concentrations. **Bi**_**2**_**O**_**2**_**Se**: (b) Q-switching output power. (c) Pulse width and pulse repetition rate. (d) Single pulse energy and peak power versus the pump power. (e) Typical pulse profile and train (the inset). **Ar-Bi**_**2**_**O**_**2**_**Se**: (f) Q-switching output power. (g) Pulse width and pulse repetition rate. (h) Single pulse energy and peak power versus the pump power. (i) Typical pulse profile and train (the inset).

Bi_2_O_2_Se and Ar-Bi_2_O_2_Se were employed as SAs for passive Q-switching NIR lasers, exhibiting significantly different performance. Figure 5(c) shows the pulse width and repetition rate of unannealed Bi_2_O_2_Se as functions of incident pump power. As the pump power increases, the pulse duration decreases from 2,178 ns to 789 ns, while the repetition rate rises from 70 kHz to 221 kHz. Simultaneously, the single-pulse energy increases from 14 nJ to 81 nJ, and the peak power grows from 6 mW to 103 mW (Figure 5(d)). Figure 5(e) displays the narrowest single-pulse shape and the corresponding pulse train at the maximum pump power of 968 mW (inset). In contrast, Ar-Bi_2_O_2_Se demonstrates superior performance. Figure 5(g) shows that the pulse duration decreases from 1,126 ns to 434 ns with increasing pump power, while the repetition rate increases from 152 kHz to 191 kHz. The shortest pulse duration of 434 ns is obtained at a pump power of 1.6 W. And, the single-pulse energy rises from 119 nJ to 198 nJ, and the peak power increases significantly from 105 mW to 457 mW (Figure 5(h)). Figure 5(i) illustrates the narrowest single-pulse shape and its corresponding burst at the maximum pump power of 1.6 W (inset). The comparative results clearly indicate that Ar-Bi_2_O_2_Se outperforms Bi_2_O_2_Se in terms of pulse duration, single-pulse energy, and peak power. This improvement is attributed to oxygen vacancy defects, which modify the material’s energy level distribution and introduce new levels that enhance photon–material interactions. This increases 1 μm light absorption, leading to higher single-pulse energy and peak power, highlighting the critical role of oxygen vacancies in optimizing Q-switching laser performance.

### 2.8 Mode-locking operation

**Figure 6:**
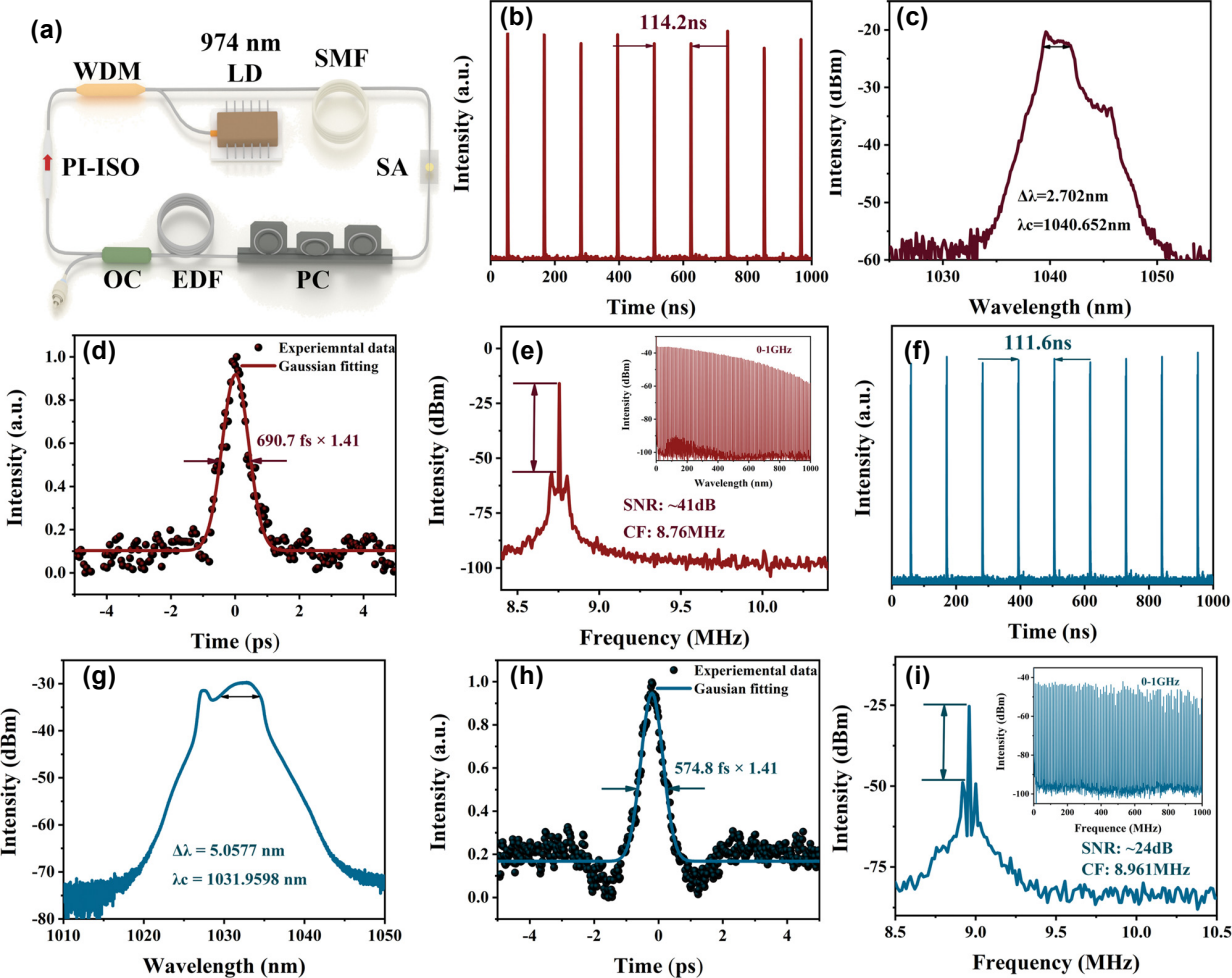
Output characteristic of Bi__2__O__2__Se and Bi__2__O__1.5__Se based mode-locked YDFL. (a) The schematic configuration of the fiber laser, **Bi**_**2**_**O**_**2**_**Se:** (b) pulse train pulse energy, (c) typical optical spectrum, (d) autocorrelation trace, (e) RF spectrum, **Ar-Bi**_**2**_**O**_**2**_**Se:** (f) pulse train pulse energy, (g) typical optical spectrum, (h) autocorrelation trace, (i) RF spectrum.

A YDFL ring cavity was meticulously assembled to facilitate the mode-locking operation at 1.5 μm. First, Bi_2_O_2_Se was deposited on a tapered fiber with a core loss of 25 % and integrated into the laser cavity, mode-locked pulses were successfully generated by carefully optimizing the pump power and polarization state. At a pump power of 150 mW, a pulse train with noise-like characteristics was observed (Figure 6(b)), with a pulse spacing of 114.2 ns, corresponding to a repetition frequency of 8.76 MHz. The optical spectrum (Figure 6(c)) had a center wavelength of 1,040.65 nm and a 3 dB spectral width of 2.70 nm. The autocorrelation trace (Figure 6(d)), fitted with a Gaussian function, revealed a pulse width of 690.7 fs. Additionally, the radio frequency (RF) spectrum displayed a signal-to-noise ratio of approximately 44 dB (Figure 6(e)), indicating high pulse stability. To evaluate the performance of annealed material, the same experimental setup was used to study Ar-Bi_2_O_2_Se and the tapered fiber loss was 24 %. Mode-locked pulses with noise-like characteristics, similar to those of Bi_2_O_2_Se, were successfully achieved by optimizing the pump power and polarization state. At a pump power of 220 mW, the pulse interval was measured as 111.6 ns, corresponding to a repetition frequency of 8.96 MHz (Figure 6(f)). The spectrum had a center wavelength of 1,031.96 nm with 3 dB spectral width of 5.06 nm (Figure 6(g)). The autocorrelation trace indicated a pulse duration of 574.8 fs (Figure 6(h)). Furthermore, the RF spectrum (Figure 6(i) and inset) showed a signal-to-noise ratio of approximately 24 dB.

Comparison of the experimental data demonstrates that Ar-Bi_2_O_2_Se not only achieves stable mode-locking but also exhibits a 2.36 nm increase in the 3 dB spectral width and a 115.9 fs reduction in pulse width. These findings validate the hypothesis that annealing enhances the nonlinear optical properties of Bi_2_O_2_Se by introducing more oxygen vacancies. These oxygen vacancies create intermediate energy levels within the bandgap, improving the material’s linear absorption while also inducing a stronger nonlinear optical response. At higher pump powers, this enhanced nonlinear effect further optimizes the material’s mode-locking behavior, enabling narrower pulse outputs. As summarized in Table 2, this study presents the performance of YDFL utilizing Bi_2_O_2_Se and Ar-Bi_2_O_2_Se as SAs and compares these results with those of lasers based on different semiconductor materials. The findings reveal that the YDFL employing Bi_2_O_2_Se and Ar-Bi_2_O_2_Se as SAs delivers output performance that is either comparable to or exceeds that of other semiconductor material-based lasers in terms of modulation depth, 3 dB spectral width, and pulse duration. Notably, while both Bi_2_O_2_S and Fe_2_O_3_ employ oxygen vacancy engineering to enhance their nonlinear optical properties, our Ar-Bi_2_O_2_Se demonstrates superior performance. Compared to Bi_2_O_2_S (which lacks precise pulse characterization due to experimental limitations), it exhibits a broader 3 dB bandwidth. Relative to Fe_2_O_3_, it achieves a stronger modulation effect, with a more significant increase in modulation depth and a greater reduction in pulse width. Our findings ultimately confirm that argon-annealing-based vacancy engineering is an effective strategy for modulating the nonlinear optical properties of Bi_2_O_2_Se and enhancing the performance of ultrafast pulsed lasers.

**Table 2:** Performance summary of mode-locked fiber lasers operating at 1 μm by using different types of materials as SAs.

Material	Center wavelength (nm)	3 dB Δ*λ* (nm)	Pulsed width (ps)	Modulation depth (%)	Ref.
Sb_2_Te_3_	1,031.7	2.5	18.8	5.20	[50]
Cr_2_S_3_	1,030.3	1.1	10,200	2.06	[51]
WS_2_	1,063.9	1.5	2,500	1.20	[52]
Fe_3_O_4_	1,072.7	0.09	456	8.50	[53]
Bi_2_Se_3_	1,085.5	0.23	46	8	[54]
Graphene-based	1,069.1	0.66	38.6	11.5	[55]
Few-Layer BP	1,064.96	1.24	7.54	5.3	[56]
Gold nanorods	1,039.0	0.05	0.84	14.5	[57]
Bi_2_O_2_S	1,031.7	0.75	–	–	[58]
Fe_2_O_3_-A	1,031.6	–	594	3.51	[59]
Fe_2_O_3_-N	1,035.8	–	550	3.05	[59]
Bi_2_O_2_Se	1,040.6	2.70	0.691	5.7	This work
Ar-Bi_2_O_2_Se	1,031.96	5.06	0.575	7.1	

## 3 Experimental section

### 3.1 Theory and computational details

We carried out the first-principles calculations based on the density functional theory (DFT) within the generalized gradient density approximation (GGA) as implemented in the Vienna ab Initio simulation package (VASP 5.4.4) [60], [61]. To treat the core issues, the projector augmented wave (PAW) pseudopotential was used. To reduce the influence of periodic boundary conditions, a 1 × 1 × 2 Bi_2_O_2_Se crystal structure with a total of 20 atoms was used for the calculation. The cutoff energy of 500 eV was used for the expansion of the plane wave. The Brillouin zone was integrated with a sufficient k-point mesh of 7 × 7 × 7 in the Γ-centered sampling technique for optimizing the geometric structure. Between two successive simulation steps, the ground state’s convergence condition was set to 10^−8^ eV. During the geometric optimization, all atoms and the cell could fully relax until the Hellmann–Feynman force acting on each atom was smaller than 0.01 eV. To address the underestimation of the band gap by the Perdew–Burke–Ernzerhof (PBE) functional, the Heyd–Scuseria–Ernzerhof (HSE06) hybrid functional was employed.

In the current research, to obtain more accurate optical properties, we performed many-body perturbation theory calculations: First, quasi-particle energies were corrected via the G0W0 approximation, followed by solving the Bethe–Salpeter equation (BSE) to account for excitonic effects. The G0W0 and BSE provide a better understanding of the quasi-particle energy and excited state characteristics of materials, offering much greater consistency with experimental spectra than DFT. Due to the extreme computational power requirements of this algorithm, the response function’s cutoff energy was set to 500 eV and the *k*-mesh grid sampling was set to Γ-centered 2 × 2 × 2. The G_0_W_0_ obtains the quasi-particle energy in a single GW iteration step, which can be expressed as [62]

(6) $$\begin{array}{ll} E_{nk}^{\left(1\right)} & =\varepsilon_{nk}+Z_{nk}^{\left(0\right)}Re\left[\left\langle \phi_{nk}\left|-\frac{\Delta }{2}+V_{\text{ext}}+V_{h}\right.\right.\right.\\ & \;+\left.\left.\left.\Sigma^{\left(0\right)}\left(\omega =\varepsilon_{nk}\right)-\varepsilon_{nk}\right|\phi_{nk}\right\rangle \right] \end{array}$$

where $E_{nk}^{\left(1\right)}$ is quasi-particle energies, *ɛ*_*nk*_ is Kohn–Sham eigenvalues, $Z_{nk}^{\left(0\right)}$ is renormalization factor, *ϕ*_*nk*_ is orbitals, Δ is band-gap, *V*_ext_ is the external potential of the nuclei, and *V*_*h*_ is Hartree potential.

In the BSE, the excitation energies correspond to the eigenvalues *ω*_*λ*_ of the following linear problem

(7) $$\begin{array}{c} \left(\begin{array}{cc} A & B\\ B^{*} & A^{*} \end{array}\right)\left(\begin{array}{c} X_{\lambda }\\ Y_{\lambda } \end{array}\right)=\omega_{\lambda }\left(\begin{array}{cc} 1 & 0\\ 0 & -1 \end{array}\right)\left(\begin{array}{c} X_{\lambda }\\ Y_{\lambda } \end{array}\right) \end{array}$$

where the matrices *A* and A* describe the resonant and antiresonant transitions between the occupied and unoccupied states. Terms *B* and B* described the coupling between resonant and antiresonant terms.

### 3.2 Material preparation

Mix high-purity Bi_2_O_3_, Se, and Bi powder evenly in an argon glove box according to the stoichiometric ratio to avoid oxidation. The mixed powder was then loaded into a quartz ampoule, evacuated to high vacuum, and sealed. The sealed ampoule was heated to 500 °C at a rate of 5 °C/min and held for 6 h. After cooling, the ampoule was opened, and the intermediate product was thoroughly ground to ensure homogeneity. The powder was then resealed in a vacuum ampoule, heated to 950 °C, and calcined for 12 h. Finally, the sample was slowly cooled to room temperature to obtain phase-pure Bi_2_O_2_Se powder.

### 3.3 Annealing treatment

Prior to annealing, the reaction chamber was purged with high-purity argon gas (99.999 %) at least three times to minimize oxygen content. Chemical vapor deposition (CVD) was used to anneal Bi_2_O_2_Se to produce oxygen vacancy defects (Figure S2). The temperature was raised to 750 °C at a rate of 5 °C/min in an argon atmosphere. Argon used as a carrier gas was maintained at a constant flow rate of 100 sccm. The sample was heated at the target temperature for 4 h and then naturally cooled to room temperature. The entire annealing process was conducted at ambient pressure without intentional oxygen introduction.

### 3.4 I-scan and open-aperture Z-scan system

The I-scan/Z-scan were performed using homemade equipment as shown in Figure S3. In the I-scan, a laser beam at a wavelength of 1 μm is split into two parts by a beam splitter. The reference beam goes directly to power meter 1, while the test beam passes through a focusing lens (*f* = 75 mm) and is directed onto the material and the transmitted beam’s intensity is then measured by power meter 2. By comparing the intensities recorded by power meters 1 and 2, the saturable absorption characteristics of the material are evaluated. In the Z-scan, the sample is precisely moved along the *Z*-axis by a motorized displacement stage and the transmitted beam’s intensity is measured by power meter 2. By analyzing the changes in the measured intensity, the nonlinear optical properties of the material can be determined.

### 3.5 Q-switched NIR lasers

For passively Q-switched operation at 1 µm, the 808 nm fiber-coupled laser diode (FAP system, Coherent Inc, USA) was used as the pump source. The resonant cavity used is a flat-flat cavity with a length of 25 mm (Figure 5(a)). The diameter of the coupled fiber was 200 μm and the numerical aperture (NA) was 0.22. In the experiment, the indium foil wrapped laser crystal was mounted into a copper heat sink, and the temperature of the crystal was kept at 12 °C by a water cooling system to fully reduce the thermal effect of the crystal. The transmittance of the output mirror was 6.8 %. To minimize heat loss, the SA was placed inside the cavity near the output coupler, and a long-pass filter was placed to filter out the residual pump light before measuring the output power.

### 3.6 Mode-locking lasers

The carefully designed YDFL ring cavity is used for mode-locking operation at the 1 μm, and the schematic diagram of the fiber laser is shown in Figure 6(a). The laser cavity adopts a traditional ring resonator structure, including 1 m of ytterbium-doped fiber (YDF, model SM-YSF-HI-HP) with a group velocity dispersion (GVD) of 26 ps^2^/km at 1,030 nm, and 22.7 m of single-mode fiber (SMF, model HI-1060) with a GVD of 22.3 ps^2^/km. The cavity also includes a polarization-independent isolator, a polarization controller (PC), and a wavelength division multiplexer (WDM) operating at 980/1,030 nm. Additionally, the cavity incorporates an optical coupler (OC) with a 10 % coupling ratio at 1,030 nm. Therefore, the total length of the fiber resonator is 23.7 m, resulting in a net positive dispersion of 0.492 ps^2^. The laser diode (LD) operates at 976 nm with a peak pump power of 450 mW. It is worth noting that, without the presence of the SA, adjusting the pump power (from 0 to 450 mW) or the polarization direction cannot generate ultrashort pulses.

## 4 Conclusions

This study demonstrates that oxygen vacancy engineering significantly enhances the linear and nonlinear optical properties of Bi_2_O_2_Se. Annealing introduces intermediate energy levels by increasing oxygen vacancies, reduce the bandgap, improve optical absorption, and strengthen the nonlinear response. Main results include an increase in modulation depth from 5.7 % to 7.1 %, a rise in the nonlinear absorption coefficient from 0.96 ± 0.16 cm MW^−1^ to 1.0 ± 0.12 cm MW^−1^, and enhanced performance in Q-switching and mode-locking lasers. For Ar-Bi_2_O_2_Se, the shortest pulse width reaches 434 ns, with a peak power of 457 mW and a single-pulse energy of 198 nJ. In mode-locking operation, the pulse duration is reduced to 574.8 fs, and 3 dB spectral width of 5.06 nm. These findings emphasize the crucial role of oxygen vacancies in optimizing the optical properties of Bi_2_O_2_Se, which will contribute to future applications in precision machining and biological imaging, and establish oxygen vacancy engineering as an effective strategy for its development. This work not only advances the fundamental understanding of defect-engineered optoelectronic materials but also provides a scalable pathway for designing high-performance photonic devices for next-generation laser technologies.

## Supplementary Material

Supplementary Material Details
