# Supplementary material for: Oxygen vacancy-driven bandgap tuning and ultrafast laser performance in Bi2O2Se
Source: Nanophotonics. 2025 Sep 12;14(20):3265–77. doi: 10.1515/nanoph-2025-0274 (PMC12588564; doi:10.1515/nanoph-2025-0274)
Supplement: Supplementary file 1 — Supplementary Material Details [file j_nanoph-2025-0274_suppl_001.docx]

Supporting Information for Oxygen Vacancy-Driven Bandgap Tuning and Ultrafast Laser Performance in Bi_2_O_2_Se

Qingling Tang, Zhongben Pan*, Zeshang Ji, Hongwei Chu, Han Pan, Dechun Li*

School of Information Science and Engineering and Key Laboratory of Laser and Infrared System of Ministry of Education, Shandong University, Qingdao 266237, PR China

* To whom correspondence may be addressed. cEmail: zhongbenpan@sdu.edu.cn; dechun@sdu.edu.cn.

**Table of contents**

[Figure S1. Two types of crystal cell diagrams (a) Bi_2_O_2_Se, (b) Bi_2_O_1.5_Se. 3](#_Toc196498184)

[Figure S2. Schematic diagram of annealing device. 3](#_Toc196498185)

[Figure S3. Schematic of I-scan/Z-scan. 4](#_Toc196498186)


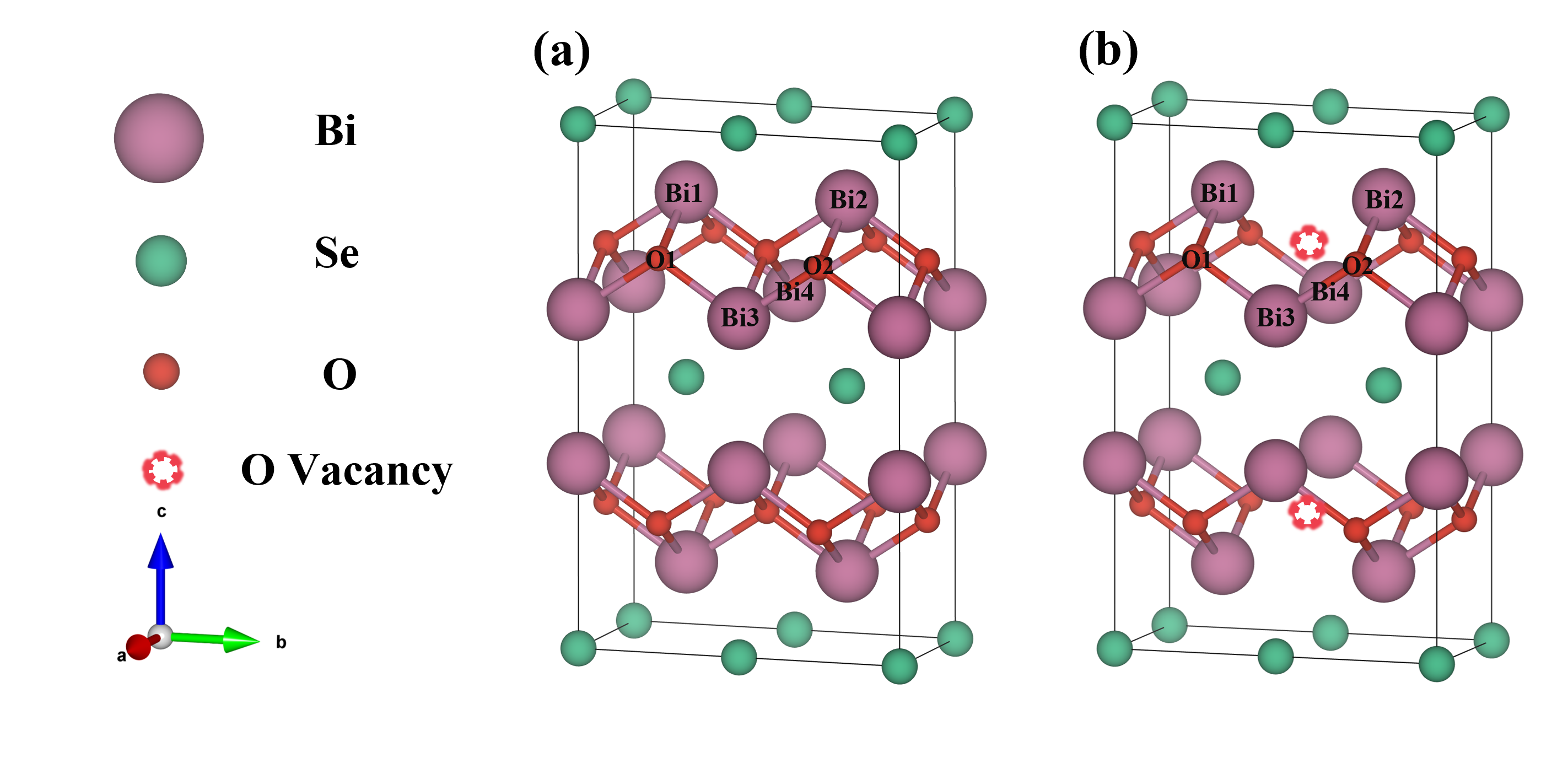


**Figure S1.** Two types of crystal cell diagrams (a) Bi_2_O_2_Se, (b) Bi_2_O_1.5_Se.


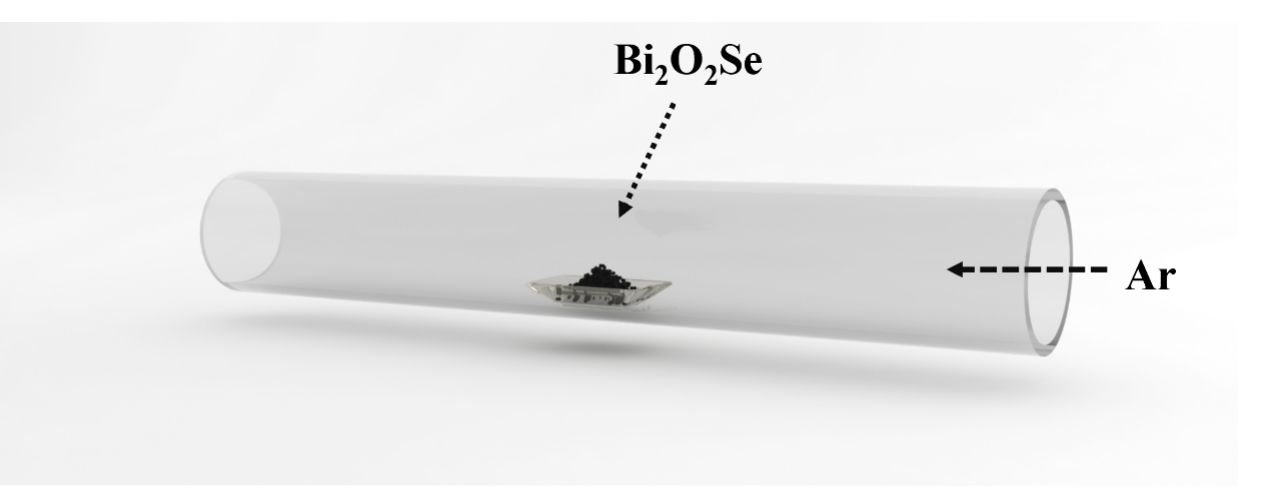


**Figure S2**. Schematic diagram of annealing device.


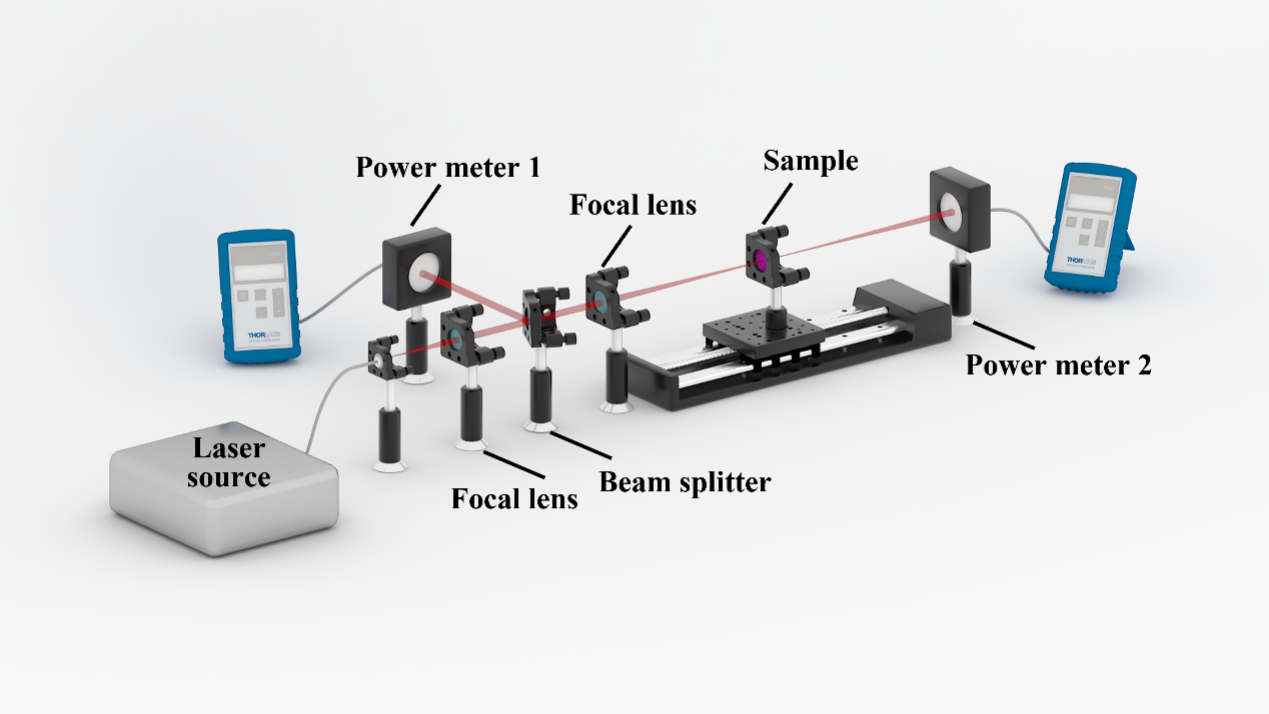


**Figure S3.** Schematic of I-scan/Z-scan.
